# Identification of GABBR2 as a diagnostic marker and its association with Aβ in Alzheimer's disease

**DOI:** 10.1016/j.bbrep.2025.102035

**Published:** 2025-04-28

**Authors:** Huijun Li, Yawei Fan, Chan Chen, Yuzhong Xu, Xiong Wang, Wei Liu

**Affiliations:** a Department of Laboratory Medicine, Tongji Hospital, Tongji Medical College, Huazhong University of Science and Technology, Wuhan, 430030, China; b Department of General Surgery, Tongji Hospital, Tongji Medical College, Huazhong University of Science and Technology, Wuhan, 430030, China; c The Baoan People's Hospital of Shenzhen, The Second Affiliated Hospital of Shenzhen University, Shenzhen, 518101, China; d Department of Public Health, Tongji Hospital, Tongji Medical College, Huazhong University of Science and Technology, Wuhan, 430030, China

**Keywords:** Alzheimer's disease, GABBR2, Diagnosis, Immune cell infiltration

## Abstract

**Background:**

Synaptic dysfunction and synapse loss occur in Alzheimer's disease (AD). The current study aimed to identify synaptic-related genes with diagnostic potential for AD.

**Methods:**

Differentially expressed genes (DEGs) were overlapped with phenotype-associated module selected through weighted gene co-expression network analysis (WGCNA), and synaptic-related genes. The overlapped hub genes were further processed using machine learning algorithms, intersected with module gene from protein-protein interaction (PPI) network constructed with DEGs, to yield co-hub genes. The diagnostic potentials of the co-hub genes were examined by receiver operating characteristic (ROC) analysis. Correlation between co-hub genes with clinical features and immune cell infiltration was analyzed. Finally, the expression of co-hub genes was analyzed in several datasets and validated in AD transgenic mice.

**Results:**

A total of three co-hub genes were identified, including MAP1B, L1CAM, and GABBR2. GABBR2 showed area under the curve (AUC) values of 0.98, 0.81, and 0.88 in the training and two external validation datasets. GABBR2 was negatively correlate with beta- and gamma-secretase activities, and infiltration of natural killer T cells and effector memory CD8 T cells. Finally, GABBR2 was validated to be downregulated in AD transgenic mice, aligning with bioinformatic findings. GABBR2 overexpression in N2a/APP cells increased ADAM10 while decreased of BACE1, leading to upregulation of sAPPα while downregulation of sAPPβ.

**Conclusion:**

In conclusion, GABBR2 acts as a novel biomarker for the diagnosis of AD and negatively correlated with Aβ in AD.

## Highlights

• DEGs were enriched in pathways of several neurodegeneration disorders.
• GABBR2 had AUC value greater than 0.8 in all three datasets.
• GABBR2 expression was significantly correlated with pathogenic features of AD.
• GABBR2 was significantly reduced in 6-month-old 5xFAD mice.

## 1 Introduction

Alzheimer's disease (AD) is common in the elderly, which will become a major global public health crisis due to the continued reduction in fertility and increased life expectancy, placing a heavy burden on healthcare systems [1]. AD is characterized by amyloid beta (Aβ) plaques due to reduced Aβ clearance or excessive production, and pathological hyperphosphorylated tau aggregates within dendrites and neuronal cell bodies, which together drive progressive synaptic dysfunction, neuronal loss, and cognitive decline [2,3]. Despite recent therapeutic advances, including FDA-approved monoclonal antibodies targeting Aβ, their safety and efficacy remain controversial [4]. Conventional tau-targeting therapies have also yielded limited success in clinical trials [5]. These challenges underscore the urgent need for novel diagnostic biomarkers and therapeutic targets in AD.

Synaptic dysfunction and synapse loss are the strongest pathological correlates of cognitive impairment in AD [6,7]. A key contributor to this process is glutamatergic excitotoxicity, where dysregulated glutamate signaling leads to neuronal hyperactivity and death [8,9]. However, neuronal function relies on the delicate excitatory/inhibitory (E/I) balance maintained by both excitatory glutamatergic and inhibitory GABAergic systems [10]. Increasing evidence suggests that disruption of GABAergic inhibition exacerbates cognitive deficits and neurodegeneration in AD [11,12]. Specifically, reductions in GABA levels and GABAergic synapses have been observed in AD brains [13], alongside diminished GABA receptor-positive neurons [14] and altered GABA receptor subtypes associated with behavioral impairments in AD models [15,16]. Furthermore, E/I imbalance contributes to network hyperexcitability and seizure susceptibility, affecting approximately 22 % of AD patients and worsening cognitive decline [17,18].

Among GABAergic components, GABA type B receptor subunit 2 (GABBR2) plays a pivotal role in modulating inhibitory neurotransmission and maintaining synaptic stability. Previous studies have reported GABBR2 downregulation in AD [19], suggesting its potential involvement in disease progression. However, its diagnostic utility and mechanistic role remain incompletely understood.

In this study, we aimed to identify synaptic-related genes with diagnostic potential in AD, focusing on GABBR2 as a candidate biomarker. By integrating differential expression analysis, weighted gene co-expression network analysis (WGCNA), protein-protein interaction (PPI) networks, and machine learning across multiple AD cohorts, we systematically screened synaptic genes associated with AD phenotypes. We further validated the diagnostic performance and mechanistic relevance of GABBR2 in AD pathogenesis.

## 2 Methods

### 2.1 Animals

The 6-month-old 5xFAD mice were purchased and maintained by Gene&Peacebiotech (JiangSu, China). Mice were housed under a 12-h light/dark cycle.

### 2.2 Materials

Anti-APP, anti-ADAM10, and anti-PS1 antibodies were purchased from Abcam (Cambridge, UK), anti-BACE1 antibody was from Cell Signaling Technology (Danvers, MA, USA), anti-sAPPα and anti-sAPPβ antibodies were from IBL (Fujioka-Shi, Gunma, Japan), anti-GAPDH and anti-GABBR2 antibodies were from Proteintech (Wuhan, China). The Aβ42 ELISA kit was purchased from Elabscience (Wuhan, China). The coding region of mouse GABBR2 (NM_001081141.2) was cloned into pCMV vector.

### 2.3 Dataset

**Fig. 1:**
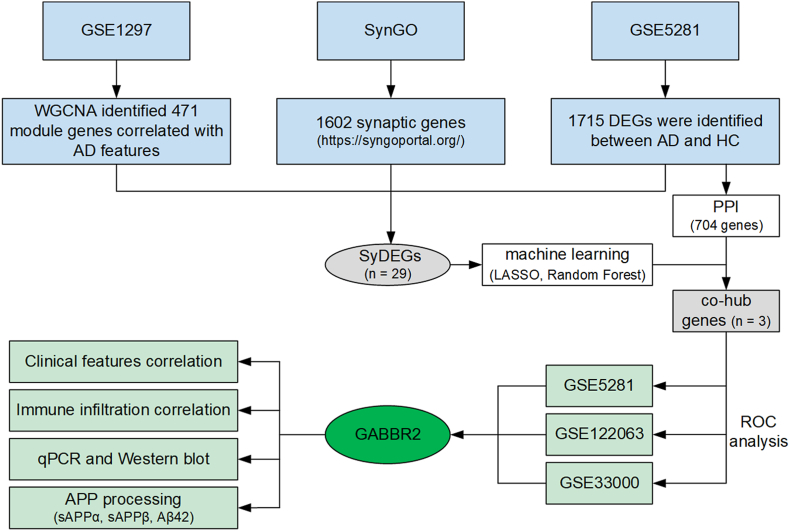
**The flowchart of this study.** AD, Alzheimer’ disease; HC, healthy control; DEGs, differentially expressed genes; WGCNA, weighted gene co-expression network analysis; PPI, protein–protein interaction; SyDEGs, synaptic-related DEGs; LASSO, least absolute shrinkage and selection operator; qPCR, real-time quantitative PCR; ROC, receiver operating characteristic curve.

The flow chart is shown in Fig. 1. All datasets used were obtained from the GEO, including GSE5281, GSE1297, GSE122063, GSE106241, and GSE33000. GSE5281 provided hippocampus gene expression matrix from 13 healthy controls and 10 AD patients. 22 AD patients with hippocampal gene expression information and clinical features, including severity (incipient, moderate, severe), age, gender, NFT count, Braak stage, and MiniMental Status Examination (MMSE) score, were included in GSE1297 dataset. Frontal cortex gene expression matrix from 22 healthy controls and 28 AD patients were collected from GSE122063 dataset. 55 AD temporal cortical samples with detailed information of Braak stage, beta secretase activity, and gamma secretase activity, were included in GSE106241 dataset. Prefrontal cortex gene expression matrix from 157 healthy controls and 310 AD patients were included in GSE33000 dataset. GSE5281 dataset was used for differentially expressed genes (DEGs) identification and ROC analysis. GSE1297 dataset was used for WGCNA. GSE122063 and GSE33000 datasets were used for ROC analysis. GSE106241 was used for the correlation with GABBR2 expression and clinical features. The expression matrix was processed with log2 transformation and normalization within the R program (version 4.3.3).

### 2.4 Identification of differentially expressed genes

The principal component analysis (PCA) is widely used to explore similarities and relationships between individual samples. The quality of tissue samples from GSE5281 dataset was assessed using the PCA with FactoMineR (version 2.9) and factoextra (version 1.0.7) R packages. The DEGs between AD patients and the healthy controls (HC) were identified using limma (version 3.57.9) R package with the ǀlog2FoldChangeǀ and adjusted *p*-value cutoff value of 1 and 0.01 respectively [20]. The expression of DEGs were displayed in volcano plot and heatmap using ggplot2 (version 3.5.0) R package. Functional annotation of these DEGs was performed by using Kyoto Encyclopedia of Genes and Genomes (KEGG) enrichment analysis with the parameter of qvalue Cutoff = 0.05.

### 2.5 Weighted gene co-expression network analysis

The WGCNA (version 1.72–1) R package was utilized to perform the WGCNA using GSE1297 dataset which included 22 AD patients with hippocampal gene expression information and clinical features. The log_2_ transformed expression matrix was used, soft thresholding power of 9 was selected, and signed R^2^ was 0.85 depicted by the mean connectivity. A hierarchical clustering tree was constructed and a total of 20 different modules were categorized with module size cut-off of 30, the cut-height cut-off of 0.25 and verbose of 3. Pearson correlation test was used to examine correlation between modules and clinical features, and the correlation coefficient and p-value were displayed in the heatmap. Functional annotation of genes within the selected module was performed using the KEGG enrichment analysis.

### 2.6 Identification of synaptic-related DEGs

A list of 1602 synaptic-related genes was downloaded from SynGO (https://syngoportal.org/). These synaptic-related genes, blue module genes in WGCNA, and DEGs from GSE5281 dataset were intersected using the jvenn diagram [21]. A total of 29 intersected genes were identified and named as SyDEGs.

### 2.7 Hub genes selection by machine learning

The machine learning procedures were widely utilized to filter variables, and both the least absolute shrinkage and selection operator (LASSO) and random forest were used. The glmnet (version 4.1–8) R package was used to conduct the LASSO analysis with the minimum λ used as the optimal value, and 5 × cross-validation was used. Random forest was performed with the randomForest (version 4.7–1.1) R package. The variable importance was ranked by both mean decrease Gini and mean decrease accuracy, and the top 7 genes ranked by the two methods were intersected as hub genes from random forest.

### 2.8 PPI network construction

The DEGs in the GSE5281 dataset was used as input to construct the PPI network using STRING and visualized with Cytoscape (version 3.8.2) software. The built-in MCODE plugin was used to further cluster the PPI network with default setting. The first module including 704 genes was selected as PPI genes, which were further intersected with hub genes selected by machine learning to obtain the co-hub genes.

### 2.9 The receiver operating characteristics analysis

The area under the receiver operating characteristic (ROC) curve (AUC) was calculated with pROC (version 1.18.5) R package, and the ROC curve was drawn by ggplot2 (version 3.5.0) R package.

### 2.10 Immune infiltration analysis

Pornpimol Charoentong et al. reported the marker genes used to define the 28 immune cell subtypes [22]. The ssGSEA was used to predict the infiltration of 28 types of immune cells using the GSVA (version 1.50.0) R package. The differences of immune cell levels were compared with Wilcoxon test. Pearson correlation between GABBR2 expression level and immune cell proportion was examined and displayed using the ggscatter function from ggpubr (version 0.6.0) R package.

### 2.11 Real-time quantitative PCR

TRIzol (Thermo Fisher Scientific, Carlsbad, CA) was used to extract Hippocampal RNA. Total RNA was converted into cDNA with the cDNA Synthesis Kit (Yeasen, Shanghai, China), and real-time quantitative PCR (qPCR) was conducted using the ABI7500 machine (Applied Biosystem, Pleasanton, CA). The following primers were used: Gabbr2 F: 5′-GTTGTGCCTTTGAGGAGAGC-3′, R: 5′-AGTCCACTCCGATGTAGCCT-3’. Gapdh, F: 5′-GGCATCTTGGGCTACACTG-3′, R: 5′-GTGGAAGAGTGGGAGTTGC-3’.

### 2.12 Western blot

20 μg protein was added to 8 % SDS-PAGE gel, and were transferred to PVDF transfer membrane (Merck Millipore, Danvers, MA). The membrane was incubated with specific antibody overnight, and further incubated with secondary antibody.

### 2.13 Statistical analysis

All data were analyzed using R program (version 4.3.3). Student's t-tests or Wilcoxon test was used to camporee the difference between groups. Significant difference was considered when the *p*-value <0.05.

## 3 Results

### 3.1 Identification of DEGs between AD and healthy subjects

**Fig. 2:**
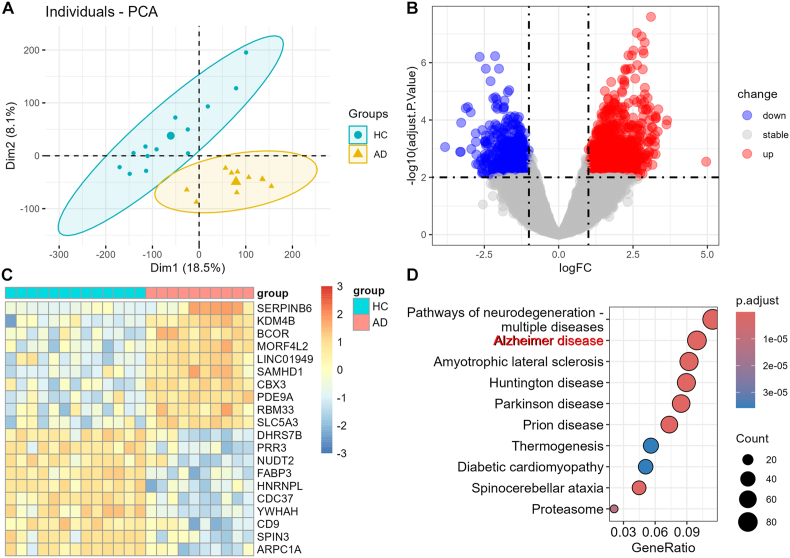
**Identification and functional annotation of differentially expressed genes.** (A) The scatter plot represented sample distribution in the PCA of the GSE5281 dataset (n = 23) using the first two components. Samples within the same group were colored by indication and ellipses. (B) Volcano plot displayed DEGs between AD and HC. Upregulated gene were colored in red, and downregulated genes were colored in blue. (C) Heatmap displayed the expression of top 10 upregulated and downregulated genes between AD and HC. (D) KEGG enrichment results of DEGs.

PCA is widely used to explore similarities and relationships between individual samples, and it revealed that samples were primarily clustered by group in the GSE5281 dataset (Fig. 2A). 1715 DEGs were identified in hippocampus between AD and healthy control (HC), including 1046 increased and 669 decreased genes (Fig. 2B). The expression of top 10 increased and decreased genes was displayed in heat map (Fig. 2C). The upregulated genes included PDE9A and SLC5A3. PDE9A is a promising target for treatment of AD [23]. SLC5A3 was found commonly upregulated in numerous datasets of AD, and was involved in peripheral nervous system development and the transport of potassium ions across plasma membranes GO terms [24]. The downregulated genes included CDC37 and FABP3, NUDT2, PRR3, and DHRS7B. CDC37 regulates tau phosphorylation and stability [25]. FABP3, a biomarker of neuronal membrane disruption, was associated with lipid dyshomeostasis in AD [26]. KEGG enrichment analysis of DEGs revealed that these DEGs were enriched in pathways of several neurodegeneration disorders, including AD (Fig. 2D). Amyotrophic lateral sclerosis, Huntington disease, Parkinson disease, and Prion disease were also enriched in DEGs.

### 3.2 Identification of specific module genes related to clinical features of AD

**Fig. 3:**
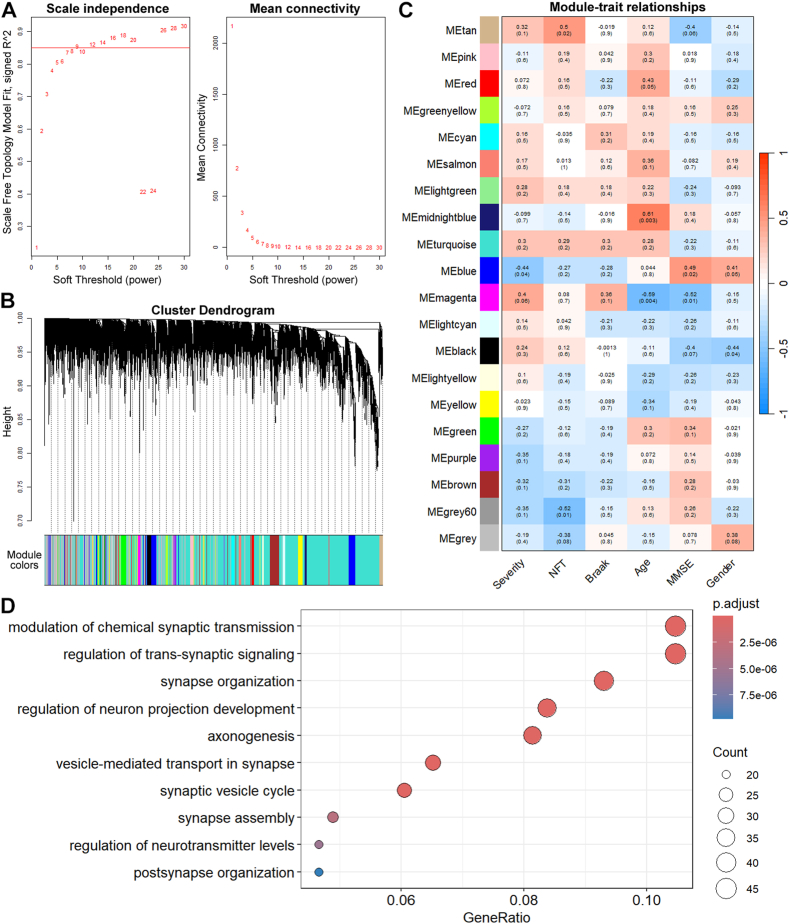
**Identification of specific module genes related to clinical features of AD.** (A) The left plot displayed the scale free topology model fit index (y-axis) for various soft-thresholding powers (x-axis). The right plot displayed the mean connectivity (y-axis) for different soft-thresholding powers (x-axis). (B) Correlated genes were grouped into colored modules. (C) Correlation between module and trait. The red and blue colors represented positive and negative correlations respectively. (D) GO biological process enrichment analysis of genes within the blue module.

WGCNA is one of the most common methods of investigating the gene co-expression network. WGCNA identifies modules consisting of genes with similar expression patterns by calculating gene expression networks. A soft-thresholding power of β = 9 (R^2^ = 0.85) was selected as the most appropriate threshold value in the WGCNA of the GSE1297 dataset including hippocampal expression data of 22 AD patients, and 20 different modules were identified (Fig. 3A and B). Pearson correlation analysis between modules and clinical traits was performed, and was displayed by a heatmap (Fig. 3C). The blue module (471 genes) displayed the highest correlation with severity and MMSE (r = −0.44, *p* = 0.04; r = 0.49, *p* = 0.02). The GO analysis highlighted synaptic activity, including synaptic vesicle cycle, synaptic transmission, and synapse organization (Fig. 3D).

### 3.3 Identification of hub genes by machine learning

**Fig. 4:**
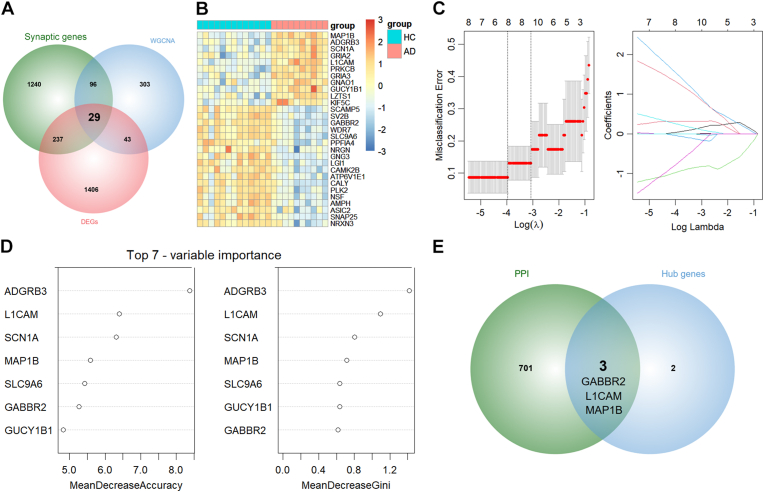
**Identification of hub genes by machine learning.** (A) Venn plot displayed the intersection of DEGs, blue module genes, and synaptic genes. (B) Heatmap displayed the expression of intersected genes between AD and HC. (C) The misclassification errors and coefficients of selected genes with various lambda values. (D) Top 7 genes ranked by mean decrease accuracy and mean decrease Gini. (E) Venn plot displayed the intersection of PPI module genes and hub genes from machine learning.

Synaptic genes were achieved from SynGO which is an evidence-based and expert-curated annotations of 1602 synaptic genes [27]. A total of 29 SyDEGs were identified by intersecting DEGs in the GSE5281 dataset, blue module genes in the GSE1297 dataset, and synaptic genes from SynGO (Fig. 4A). The heatmap revealed that 11 and 18 SyDEGs were upregulated and downregulated respectively (Fig. 4B). The machine learning procedures were utilized to determine hub genes with diagnostic potential. Eight genes were selected using LASSO analysis (Fig. 4C). Common top 7 genes ranked by mean decrease accuracy and mean decrease Gini were selected in random forest analysis (Fig. 4D). Five hub genes were intersected from LASSO and random forest, including ADGRB3, SLC9A6, MAP1B, L1CAM, and GABBR2. The PPI network of DEGs in the GSE5281 dataset was constructed. The MCODE plug was applied to identify gene modules, and the first module including 704 genes was selected. Finally, three co-hub genes (MAP1B, L1CAM, and GABBR2) were identified by intersecting the PPI genes and hub gene from machine learning (Fig. 4E).

### 3.4 Diagnostic potential of co-hub genes for AD

**Fig. 5:**
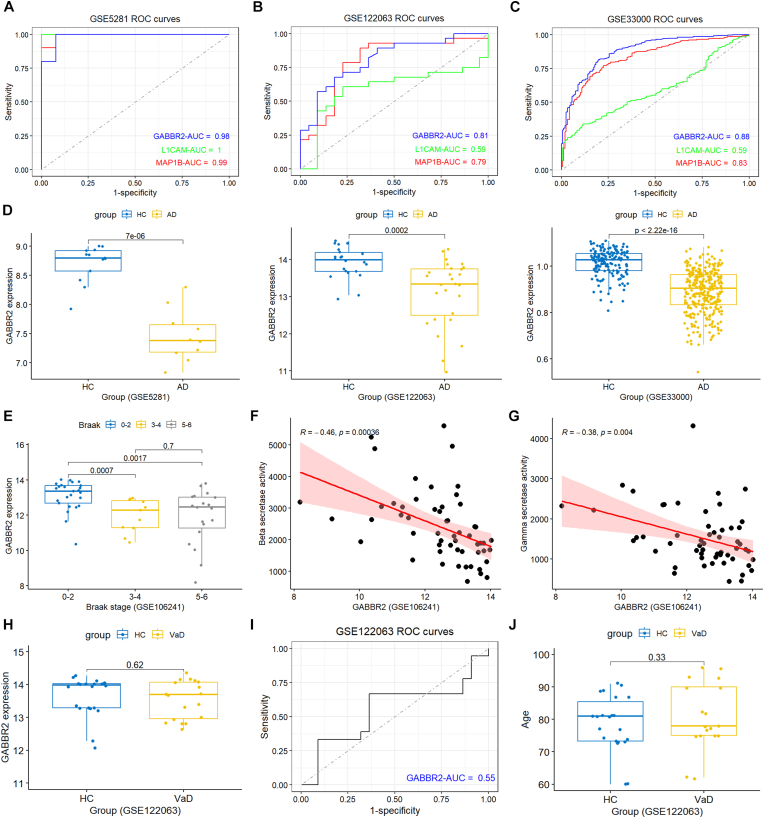
**Diagnostic potential of co-hub genes for AD and clinical correlation of GABBR2.** (A) ROC curves of the three co-hub genes (GABBR2, MAP1B, L1CAM) in the training dataset GSE5281. (B) ROC curves of co-hub genes in the validation dataset GSE122063 (frontal cortex samples). (C) ROC curves of co-hub genes in the validation dataset GSE33000 (prefrontal cortex samples). (D) Expression levels of GABBR2 in AD and healthy controls across GSE5281, GSE122063, and GSE33000 datasets. (E) GABBR2 expression across different Braak stages in the GSE106241 dataset, showing significant downregulation in stages 3–4 and 5–6 compared with stages 0–2. (F) Pearson correlation between GABBR2 expression and β-secretase activity in GSE106241. (G) Pearson correlation between GABBR2 expression and γ-secretase activity in GSE106241. (H) Boxplot showing GABBR2 expression in temporal cortex samples from HC (n = 22) and vascular dementia (VaD) patients (n = 18) in the GSE122063 dataset. (I) ROC curve analysis of GABBR2 for distinguishing VaD from HC in GSE122063. (J) Boxplot comparing age between HC and VaD groups in GSE122063.

The AUC values of three co-hub genes were all greater than 0.95 in the training GSE5281 dataset (Fig. 5A). In the validation GSE122063 dataset including 22 healthy and 28 AD frontal cortex samples, only GABBR2 and MAP1B had AUC values greater than 0.78 (Fig. 5B). We further validated in GSE33000 dataset which included 157 healthy and 310 AD prefrontal cortex samples, and the result revealed that GABBR2 and MAP1B had AUC values greater than 0.82 (Fig. 5C). GABBR2 had AUC value greater than 0.8 in all three datasets, and it was further investigated. The expression of GABBR2 was substantially reduced in AD patients compared with healthy controls in all three datasets (Fig. 5D).

The correlation between GABBR2 and clinical features of AD patients was also examined. The GSE106241 dataset included the gene expression in temporal cortical tissue samples, Braak stage, beta and gamma secretase activities of 55 AD patients. Tau pathology could be divided into six Braak stages, correlated with severity of cognitive impairment in AD patients [28]. GABBR2 was remarkably decreased in Braak stage 5–6 and stage 3–4 compared with Braak stage 0–2 (Fig. 5E), highlighting its correlation with Tau pathology. Pearson correlation analysis revealed that the expression of GABBR2 was remarkably negatively correlated with both beta- and gamma-secretase activities (Fig. 5F and G). These results indicate the close correlation of GABBR2 expression and pathogenic features of AD.

Given the clinical overlap between vascular dementia (VaD) and AD, GABBR2 expression was specifically evaluated in VaD. Gene expression profiles from the temporal cortex of 22 HC and 18 VaD patients in the GSE122063 dataset were analyzed. The expression of GABBR2 showed no significant difference between VaD and HC (Fig. 5H). An AUC of 0.55 was obtained from ROC curve analysis, indicating limited diagnostic value in VaD (Fig. 5I). No significant difference in age was observed between HC and VaD groups (Fig. 5J). These findings suggest that GABBR2 downregulation is specific to AD and not observed in VaD, supporting its potential as an AD biomarker.

### 3.5 Correlation between GABBR2 expression and immune cell infiltration

**Fig. 6:**
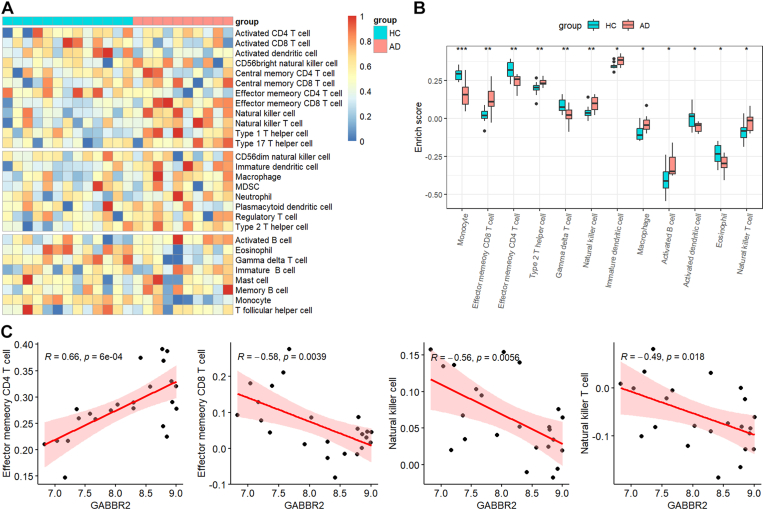
**Correlation between GABBR2 expression and immune cell infiltration.** (A) Heatmap displayed the relative abundance of immune cell infiltration across all samples. (B) Boxplot displayed the differences of immune cell infiltration between AD and HC. (C) Correlation scatter displayed the correlation between GABBR2 expression and effector memory CD4 T cells, effector memory CD8 T cells, natural killer cell, and natural killer T cells. ∗*p*-value <0.05, ∗∗*p*-value <0.01, ∗∗∗*p*-value <0.001.

Brain homing of peripheral immune cells were frequently observed in AD [29,30]. The signature genes of different immune cells were reported previously [22]. The ssGSEA method was applied to predict the infiltration of 28 types of immune cells using the GSVA (version 1.50.0) R package, and the result was displayed in heatmap (Fig. 6A). A total of 12 types of immune cells displayed significant infiltration levels between healthy controls and AD patients (Fig. 6B), including natural killer cells, effector memory CD8 T cells, macrophages, and effector memory CD4 T cells. The expression of GABBR2 was positively correlated with effector memory CD4 T cells, while was negatively correlated with effector memory CD8 T cells, natural killer cells, and natural killer T cells (Fig. 6C).

### 3.6 Validation of GABBR2 in AD animal model

**Fig. 7:**
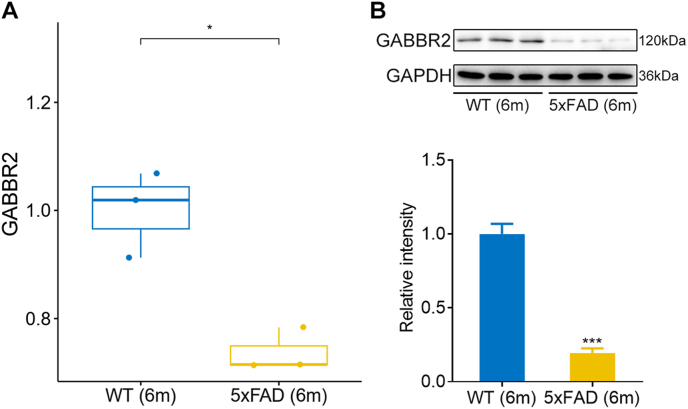
**Validation of GABBR2 in AD animal model.** (A) Expression of GABBR2 in the hippocampus of 6-month-old wild type (WD) and 5xFAD mice was detected by qPCR. (B) The protein levels of GABBR2 in the hippocampus of 6-month-old wild type (WD) and 5xFAD mice was detected by Western blot. ∗*p*-value <0.05, ∗∗∗*p*-value <0.001.

Finally, the expression of GABBR2 were further validated in the hippocampus of 5xFAD transgenic mice which expressed human APP and PSEN1 genes with five AD-linked mutations. Both the mRNA and protein levels of GABBR2 were substantially reduced in 6-month-old 5xFAD mice (Fig. 7A and B).

**Fig. 8:**
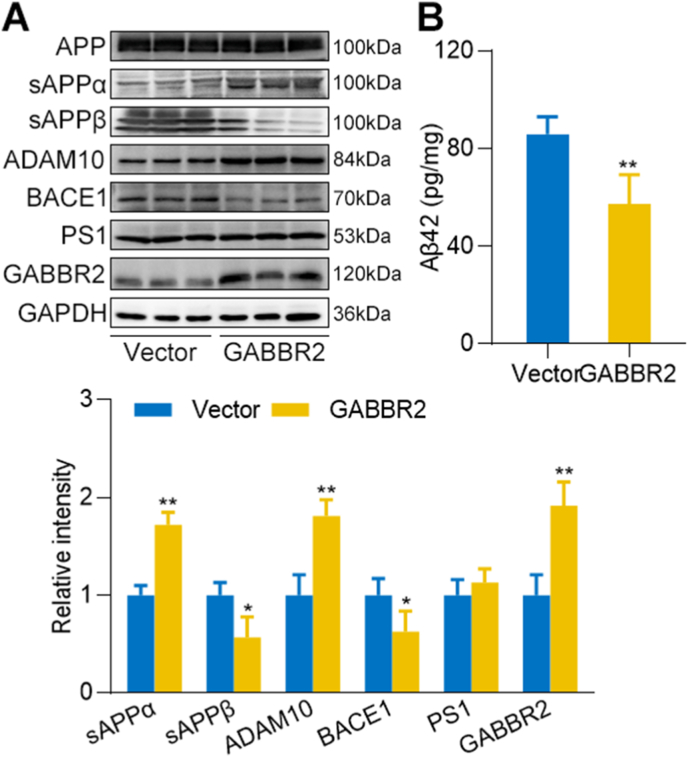
**GABBR2 influences APP processing.** (A) N2a/APP cells which stably expressed APP were transfected with GABBR2 overexpression or vector plasmid. Western blot was used to detect the protein levels of α-, β-, and γ-secretases involved in APP processing. (B) ELISA was used to detect Aβ42 level. ∗*p*-value <0.05, ∗∗*p*-value <0.01.

We further explored how GABBR2 influences amyloid precursor protein (APP) processing through its involvement in the regulation of α-, β-, and γ-secretase activities. Notably, we observed that GABBR2 overexpression in N2a/APP cells, which stably expressed APP, led to the upregulation of ADAM10 and downregulation of BACE1. This resulted in an increase in the protein level of sAPPα, while the level of sAPPβ and Aβ42 decreased (Fig. 8A and B). These findings suggest that GABBR2 may play a protective role in AD by modulating the non-amyloidogenic pathway of APP processing, which is mediated by ADAM10, thereby reducing the amyloidogenic pathway that involves BACE1.

## 4 Discussion

AD characteristically begins with a particularly pure cognitive impairment of memory in its earliest clinical stage, and synapse loss is one of the central features of AD. Synapse pathology in AD involves Aβ, tau, mitochondria, glial cells, and AD risk genes. AD has been recognized as synaptic disorder [31,32]. Synapse loss occurs around amyloid plaques, and soluble Aβ disrupts synaptic plasticity by acting at the postsynaptic density [33]. Aβ interacts with numerous synaptic receptors, including NMDA, mGluR5, EphB2, and EphA4, leading to intracellular Ca^2+^ overload and synaptic deterioration [32]. Normal tau binds and stabilizes axonal microtubules, while hyperphosphorylated tau aggregates at synaptic terminals and neuronal cell bodies in AD brains [34]. Tau interacts synaptogyrin 3 and impairs vesicle release in presynaptic terminal, and interacts with PSD95, disrupting glutamate receptor signaling, trafficking, reducing receptor surface expression in postsynaptic terminal [35]. In addition to glutamate activity defect, GABAergic activity is also impaired in synaptosomes from AD brain [36]. Oligomeric Aβ preferentially bound to excitatory compared with inhibitory synapses [37]. The E/I imbalance occurs in AD and contributes to cognitive impairment in AD [18]. Underlying the mechanism of synaptic GABAergic homeostasis dysfunction is essential for the illustration of AD pathophysiology. Here, our study reveals that the GABA receptor GABBR2, essential for the regulation of inhibitory synaptic transmission, was remarkably decreased in AD patients, negatively correlated with beta- and gamma-secretase activities, and displayed great diagnostic potential with AUC >0.8 in several datasets. Moreover, both transcriptional and protein levels of GABBR2 were substantially downregulated in AD transgenic mice.

Twenty-nine SyDEGs were retrieved by intersecting DEGs, blue module genes, and synaptic gene list. Subsequently, these SyDEGs were pinpointed using the LASSO and Random Forest algorithms, followed by overlapping with PPI network constructed using DEGs. Eventually, three co-hub genes were obtained, including MAP1B, L1CAM, and GABBR2. GABBR2 displayed high diagnostic accuracy in the training dataset with an AUC of 0.98, and had AUCs of 0.81 and 0.88 in two external validation datasets. Moreover, the expression of GABBR2 showed no significant difference between VaD and controls. The ROC curve indicated limited diagnostic value in VaD. These findings suggest that GABBR2 downregulation is specific to AD and not observed in VaD, supporting its potential as an AD biomarker. Although three co-hub genes (GABBR2, MAP1B, and L1CAM) were identified, GABBR2 was prioritized due to its consistent diagnostic performance across datasets, specificity to AD, and its critical role in inhibitory neurotransmission and E/I balance. In contrast, MAP1B and L1CAM showed less stable diagnostic accuracy in external validation cohorts and are more associated with neuronal development, rather than direct synaptic inhibitory regulation in AD. A consistent reduction of GABBR2 expression was found in AD patients across three different datasets. The mRNA level of GABBR2 was decreased in temporal gyrus of AD patient brains [12]. Both qPCR and Western blot were used to assess the expression of GABBR2 in the hippocampal tissues of 5xFAD transgenic mice. Consistent with the results from bioinformatic analysis from different datasets, both transcriptional and protein levels of GABBR2 were significantly decreased in 5xFAD transgenic mice. Moreover, GABBR2 was decreased in Braak stages 3–4, stages referring to mild cognitive impairment, compared with stages 0–2. Moreover, the expression of GABBR2 was negatively correlated both beta- and gamma -secretase activities. GABBR2 is essential for coupling the GABA_B_ receptor heterodimer to downstream effectors [38]. Previous study has demonstrated GABBR2 overexpression in early AD models, suggesting a compensatory response that may initially contribute to neuronal stress through calcium dysregulation, oxidative stress, or mitochondrial dysfunction [39]. However, as AD pathology progresses, cumulative synaptic loss and neurodegeneration may lead to GABBR2 downregulation, as observed in our study. This stage-dependent regulation aligns with the progressive nature of AD, where early compensatory mechanisms eventually fail.

Activation of GABBR2 subunits alleviates the anxiety-like behaviors by promoting the BDNF signaling pathway and reversing the surface expression of Kir3 channel surface expressions in rat hippocampus [40]. In addition to its role in neurons, endothelial GABBR2 promoted angiogenesis by regulating the glycolysis pathway in post-ischemia model [41]. Collectively, our findings indicate the early diagnostic potential of GABBR2 for AD, and the underlying mechanisms may involve APP processing and Aβ generation.

Brain homing of the peripheral immune cells have been frequently reported [30,42]. Effector memory CD8 T cells, type2 T helper cells, natural killer cells, macrophages, natural killer T cells, and activated B cells were increased in AD patients, while monocytes, gamma delta T cells, activated dendritic cells, and eosinophils were decreased. Moreover, positive correlation between GABBR2 and effector memory CD4 T cells was observed, while negative correlation between GABBR2 expression and natural killer T cells, natural killer cells, and effector memory CD8 T cells was observed. Effector memory CD4 T cells were correlated with memory impairment in AD patients [43]. Brain infiltrated CD8 T cells contributed to neuronal dysfunction via modulating synaptic plasticity [44]. Depletion of NK cells improved memory deficiency in 3xTg AD transgenic mice [45]. These results highlight the potential interplay between GABBR2 and immune cells in the regulation of AD progression. The downregulation of GABBR2 may result from both synaptic dysfunction due to Aβ deposition and immune-mediated neuroinflammation, forming a pathogenic feedback loop.

Although ssGSEA based on bulk RNA-seq provides insights into immune infiltration, it cannot distinguish cell-type-specific changes or fully represent brain immune profiles. Future studies using single-cell RNA sequencing or spatial transcriptomics are needed to refine these associations and validate GABBR2's role in neuroimmune interactions in AD. Our analysis, based on bulk transcriptomic data, reflects averaged gene expression across cell types and cannot distinguish cell-type-specific changes in GABBR2. Future studies using single-cell RNA sequencing or spatial transcriptomics are needed to clarify the cellular sources and patterns of GABBR2 dysregulation in AD.

In N2a/APP cells, we observed that GABBR2 overexpression led to the upregulation of ADAM10 and downregulation of BACE1. This resulted in an increase in the protein level of sAPPα, while the level of sAPPβ decreased. ADAM10, a key enzyme in the non-amyloidogenic pathway of APP processing, is regulated by transcription factors such as CREB and NF-κB [46,47]. The cAMP/PKA signaling pathway is known to activate CREB by phosphorylation, leading to increased ADAM10 expression. Recent study has shown that copper chelators could upregulate ADAM10 expression through the cAMP/PKA/CREB pathway, promoting the non-amyloidogenic cleavage of APP and improving cognitive function in APP/PS1 transgenic mice [46]. Feng Li et al. reported that l-lactate activated GABBR2, which increased neuronal cAMP levels and promoted axonal regeneration [48]. It is plausible that GABBR2 may upregulate ADAM10 expression via the cAMP/PKA/CREB pathway.

In conclusion, our study suggests that GABBR2 has the early diagnostic potential for AD. Downregulation of GABBR2 may contribute to AD progression via modulating APP processing and immune cell infiltration and functions in the brain.

## CRediT authorship contribution statement

Xiong Wang: Writing – original draft, Visualization, Validation, Investigation. Yuzhong Xu: Investigation, Conceptualization. Huijun Li: Data curation, Methodology. Yawei Fan: Data curation, Methodology. Chan Chen: Data curation. Wei Liu: Visualization, Validation, Investigation.

## Ethics approval and consent to participate

All animal experimental protocols were approved by the guidelines of the Institutional Animal Care and Use Committee of Tongji Hospital, Tongji Medical College, Huazhong University of Science and Technology. All experiments were performed in accordance with guidelines and regulations.

## Consent for publication

Not applicable.

## Availability of data and materials

The data that support the findings of this study are available from the corresponding author upon reasonable request.

## Funding

This study was supported by the 10.13039/501100003819Natural Science Foundation of Hubei Province (2022CFB150 to Huijun Li), and Health and Medical Scientific Research Project of Shenzhen Bao'an Medical Association (BAYXH2024001 to Yuzhong Xu).

## Declaration of competing interest

The authors have no conflicts of interest to declare.
