# Supplementary material for: Identification of GABBR2 as a diagnostic marker and its association with Aβ in Alzheimer's disease
Source: Biochem Biophys Rep. 2025 Apr 28;42:102035. doi: 10.1016/j.bbrep.2025.102035 (PMC12415974; doi:10.1016/j.bbrep.2025.102035)

GABBR2 in Figure 7 GAPDH in Figure 7


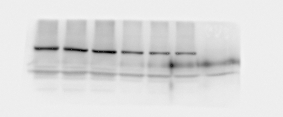

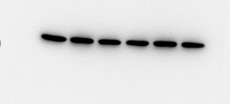


APP in Figure 8 sAPPa in Figure 8 sAPPb in Figure 8


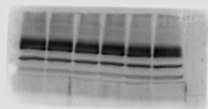

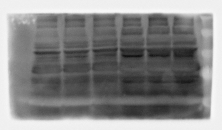


ADAM10 in Figure 8 BACE1 in Figure 8 PS1 in Figure 8


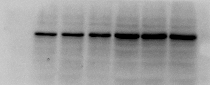

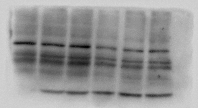

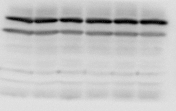


GABBR2 in Figure 8 GAPDH in Figure 8


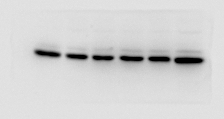

Supplement: Multimedia component 1 [file mmc1.docx]
